# Artificial intelligence algorithm comparison and ranking for weight prediction in sheep

**DOI:** 10.1038/s41598-023-40528-4

**Published:** 2023-08-15

**Authors:** Ambreen Hamadani, Nazir Ahmad Ganai

**Affiliations:** 1 National Institute of Technology, Srinagar, India; 2 Sher-e-Kashmir University of Agricultural Sciences and Technology of Kashmir, Kashmir, India

**Keywords:** Computational biology and bioinformatics, Animal breeding

## Abstract

In a rapidly transforming world, farm data is growing exponentially. Realizing the importance of this data, researchers are looking for new solutions to analyse this data and make farming predictions. Artificial Intelligence, with its capacity to handle big data is rapidly becoming popular. In addition, it can also handle non-linear, noisy data and is not limited by the conditions required for conventional data analysis. This study was therefore undertaken to compare the most popular machine learning (ML) algorithms and rank them as per their ability to make predictions on sheep farm data spanning 11 years. Data was cleaned and prepared was done before analysis. Winsorization was done for outlier removal. Principal component analysis (PCA) and feature selection (FS) were done and based on that, three datasets were created viz. PCA (wherein only PCA was used), PCA+ FS (both techniques used for dimensionality reduction), and FS (only feature selection used) bodyweight prediction. Among the 11 ML algorithms that were evaluated, the correlations between true and predicted values for MARS algorithm, Bayesian ridge regression, Ridge regression, Support Vector Machines, Gradient boosting algorithm, Random forests, XgBoost algorithm, Artificial neural networks, Classification and regression trees, Polynomial regression, K nearest neighbours and Genetic Algorithms were 0.993, 0.992, 0.991, 0.991, 0.991, 0.99, 0.99, 0.984, 0.984, 0.957, 0.949, 0.734 respectively for bodyweights. The top five algorithms for the prediction of bodyweights, were MARS, Bayesian ridge regression, Ridge regression, Support Vector Machines and Gradient boosting algorithm. A total of 12 machine learning models were developed for the prediction of bodyweights in sheep in the present study. It may be said that machine learning techniques can perform predictions with reasonable accuracies and can thus help in drawing inferences and making futuristic predictions on farms for their economic prosperity, performance improvement and subsequently food security.

## Introduction

The world population by 2050 is projected to increase to 9.9 billion and the global demand for various meat and animal products is set to increase by over 70% in the next few decades^1^. Therefore, there is a dire need to increase food production by 2050 by intensifying production on almost the same amount of land and while using the same resources. This puts pressure on the animal husbandry sector as well because, there now is a need to produce more animals using the limited land, water, and all other natural resources. It means that we need to find new and innovative approaches to produce more food which is a huge challenge for animal scientists despite a vast genetic wealth^2,3^. To address this, new technologies are are being adopted on animal farms which are evolving from traditional to high-tech^4^. Farming operations are now becoming more and more automated and the use of sensors is increasing in all aspects of farm management. This is not just reducing drudgery and labour but is also leading to an exponential increase in the amount of data generated on a daily basis. All this is leading to an exponential increase in farm data. The traditional methods and conventional strategies are not quite able to keep up with this enormous data, which is resulting in declining trends of production, especially in developing countries^5–10^.

As artificial intelligence is transforming all industries in a big way, it offers solutions to the analytic problems of animal husbandry and veterinary sciences^11^. These would help in proving many aspects of farm management which are important for reducing mortality and improving productivity^12^. They cannot just efficiently handle data but can also draw inferences that were hitherto unknown because ML techniques possess capabilities that are not present in conventional techniques. The modelling tolerance of such methods is considerably higher than statistical methodologies. This is because there is no requirement for assumptions or hypothesis testing in ML. In addition, ML benefits like the capability of handling non-linear, imprecise, noisy data. All this makes this area of science much more flexible than conventional statistical models.

The use of artificial intelligence for farming practices is rapidly becoming popular. However, the studies comparing the most popular supervised learning algorithms and ranking them are still scanty. Research for the comparison of various machine learning techniques in animal sciences for the prediction of disease^11^, performance^13^, hatchability^14^ lactation^15^, genetic merits^12,16–18^, body weights^19^, disease diagnosis^20^ and predictions^21,22^, immunity^23^ and even in molecular studies like transcriptomics^24^, RNA Sequencing gene expression^25^, genetic selection^26^ etc. For all the studies stated, algorithms like artificial neural networks, Support Vector Machines, K-nearest neighbours etc have been found to be very useful and in most cases better than the conventional approaches due to the large amount of data.

Scientists have reported multiple algorithms to be promising for solving various problems in animal sciences. The prediction of future performance is one crucial area which, if done accurately could help in making important decisions for improving both production as well as income. This study was therefore undertaken to compare the most popular ML algorithms and rank them as per their ability to make predictions on sheep farm data. An attempt was also made to fine-tune the models so that deploy-able models could be developed.

## Results

### Missing values and dimensionality reduction

**Figure 1:**
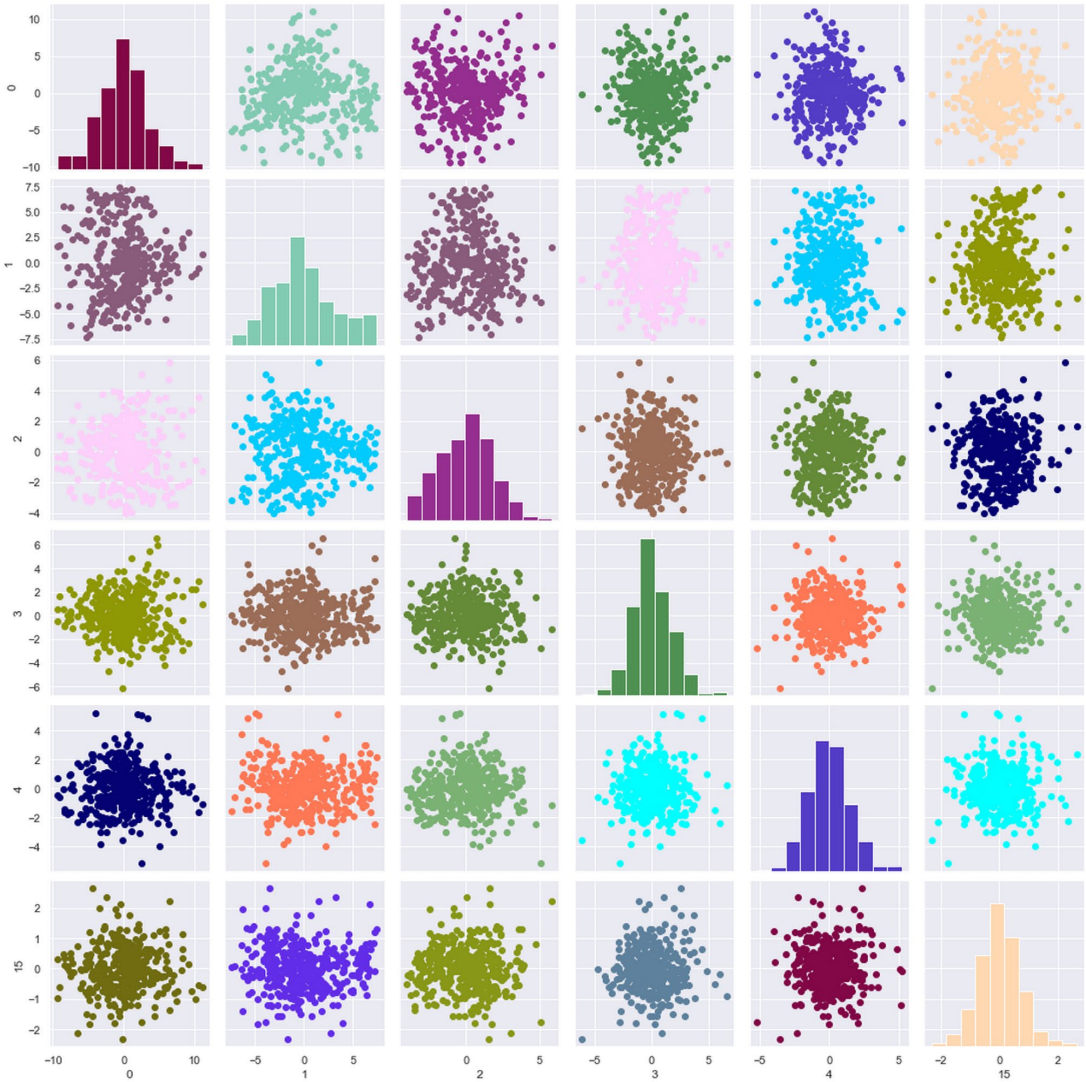
Pair plot for multicollinearity for the PCA+ FS dataset.

Our results indicated that imputation effectively removed the missing values in the dataset. Considering all variables for the dataset (for the prediction of body weight) having variance above 95% in the principal component analysis, a total of 23 features were retrieved to create the PCA dataset. The FS dataset was created by using features in the original dataset having F scores greater than 10. This way, the number of features within the FS dataset was 28. For the dataset containing features selected after PCA, 6 features were selected for the final dataset (PCA and FS) having scores greater than 4. The scores for the first 6 principal components were 1357.04, 29.97, 20.24, 13.68, 11.68, and 4.29. The multicollinearity was effectively reduced by PCA. The pair plot for multicollinearity for the PCA+ FS dataset for body weight is given in Fig. 1.

### Bayesian ridge regression and ridge regression

For Bayesian ridge regression, the RMSE, MAE, coefficient of determination and correlation coefficient for the PCA dataset was 1.084, 0.872, 0.940 and 0.979, and for the FS dataset were 0.926, 0.816, 0.957, 0.992 and for the FCA + FS dataset, they were 1.179, 0.93, 0.923 and 0.974. For ridge regression, the RMSE, MAE, coefficient of determination and correlation coefficient for the PCA dataset were 1.082, 0.871, 0.940 and 0.979, and for the FS dataset were 0.939, 0.822, 0.955, 0.991 and for the FCA+FS dataset, they were 1.178, 0.930, 0.924, 0.974 respectively. The results obtained by the Bayesian ridge regression and ridge regression were very similar. The FS dataset had the highest correlation coefficient.

**Figure 2:**
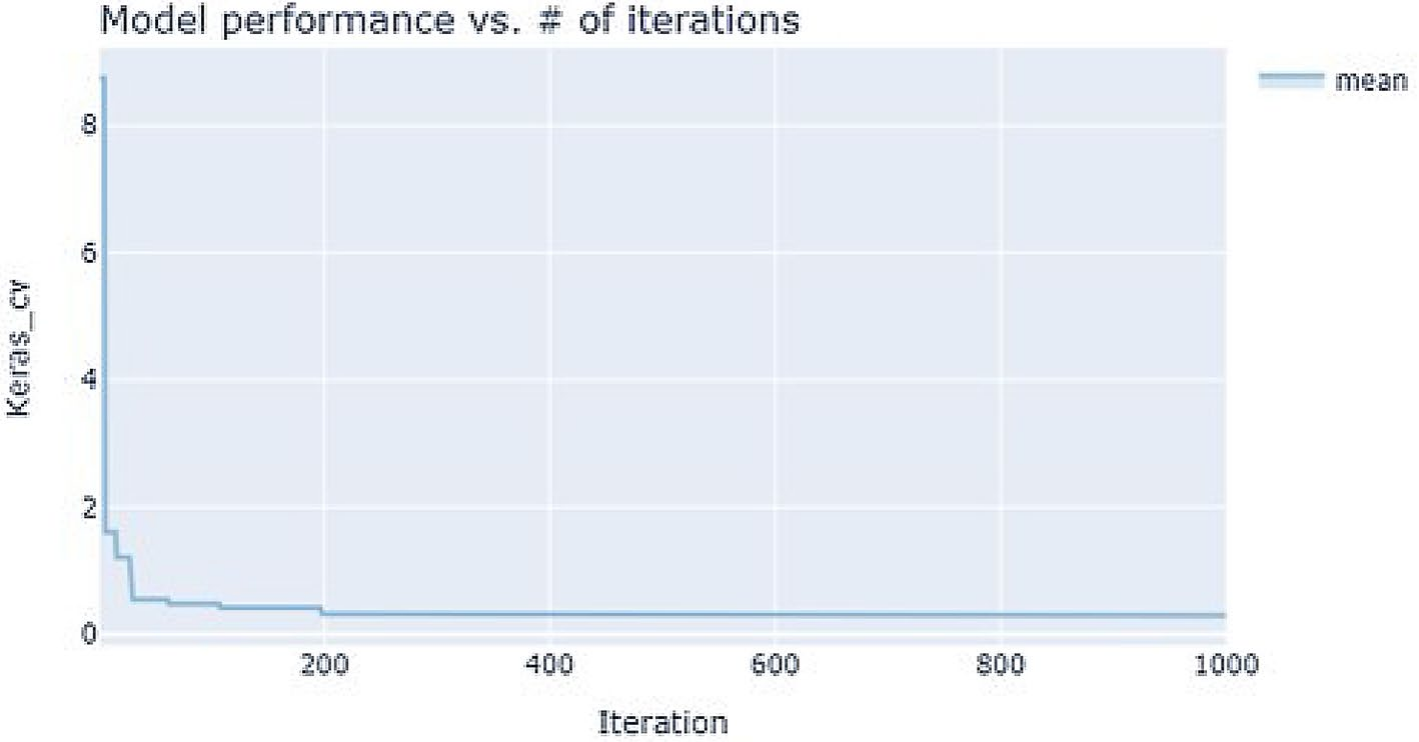
Hyperparameter optimization graph for 1000 iterations.

### Artificial neural networks

The hyperparameter optimization graph for a thousand iterations is given in Fig. 2. The results of the training of ANNs are given in Table 1. Our results indicated that the PCA+FS dataset converged earlier than the other two datasets. The results obtained by hyperparameter optimization were further refined heuristically and through this, the models could not be improved anymore. From this one may infer that the application of good searching algorithms, in this case, was enough to obtain optimum results. Out of the three datasets, the PCA dataset showed the highest correlation coefficient of 0.977. This dataset also had the highest number of neurons per layer This dataset also showed the lowest MSE, MAE, and loss when compared to the other datasets. The FS dataset alone performed better than the PCA+FS dataset and PCA dataset. The reduction in the number of features in this dataset was not enough to achieve the highest predictive ability of this dataset. The search results yielded the sigmoid activation function as well as a low learning rate as the most appropriate one for the prediction of body weights. For the hyperparameter tuning, stochastic gradient descent (sgd) and Adam both performed well as optimizers. For the activation function, ReLU and sigmoid both performed better than the rest. Of the hyperparameters trained, ReLU (rectified linear unit) and Adam (adaptive moment estimation) were the best optimizers and activation functions respectively. The number of hidden layers was 9 for all the tree models after the application of genetic algorithms. With the increase in the number of iterations, the correlation coefficient also increased. It was also seen that the more the number of iterations, the higher the correlation coefficients.

**Table 1:** Training results for artificial neural networks for prediction of body weights.

	FS	PCA + FS	PCA	DM	FS	PCA +FS	PCA
	Hyperparameter optimization				Hyperparameter optimization plus heuristic modelling		
Learning rate	0.002	0.007	0.003	0.001	0.009	0.007	0.001
Dropout rate	0.043	0.016	0.014	0.061	–	–	–
Num hidden layers	1	2	1	5	2	3	1
Neurons per layer	399	136	360	58	130	240 (2layers) 100	200
Batch size	8	30	16	8	20	20	16
Activation	sigmoid	sigmoid	sigmoid	sigmoid	ReLU	ReLU	sigmoid
Optimizer	adam	adam	sgd	rms	sgd	adam	adam (decay = 0.019)
Keras cv	0.141	0.398	0.294	6.057	–	–	–
Epoch	28/1000	100/100	81/100	49/100	55/1000	122/1000	660/1000
Validation MSE	0.132	0.338	0.339	5.991	0.442	0.498	0.377
Validation MAE	0.244	0.444	0.428	1.72	0.347	0.399	0.288
Testing MSE	0.971	1.347	1.511	4.987	1.351	1.356	1.258
Testing MAE	0.97	1.35	1.51	4.99	0.82	0.83	0.77
Test correlation	**0.984**	0.973	0.978	0.667	0.975	0.966	0.977
Test RMSE	**0.985**	1.161	1.229	2.233	1.162	1.165	1.12

The highest values obtained are in bold.

### Genetic algorithms

Genetic algorithms were sufficiently able to predict the bodyweights of sheep, but less efficiently than the other algorithms. The prediction power of genetic algorithms was the lowest among all trained algorithms for body weight prediction. Among the three (PCA, PCA+FS as well as FS) datasets for body weight prediction, the PCA+FS dataset yielded the highest correlation coefficient between true and predicted breeding values. The number of generations, fitness threshold, pop size, activation mutation rate, RMSE, MAE, R^2^, and correlation coefficients for the PCA dataset were 100, 0.980, 300, 0.001, 1.930, 1.248, 0.835, 0.874, FS + PCA dataset were 100, 0.980, 300, 0.001, 1.322, 1.031, 0.917 and 0.944 while for the FS dataset it was 100, 0.980, 300, 0.001, 1.363, 1.036, 0.929 and 0.940 respectively. The best model evolved using genetic algorithms had the number of generations, fitness threshold, population size, activation mutate rate RMSE, MAE, R^2^, and correlation coefficient of 100, 0.980, 300, 0.001, 1.322, 1.031, and 0.917.

### Support vector machines

The FS dataset had the highest correlation coefficient with the test labels the hyperparameters for which the grid search was performed. The hyperparameters for the same were ‘C’: 1000, ‘gamma’: 1, and ‘kernel’: ‘linear’. Table 2 gives the results obtained from training and testing this algorithm. The linear kernel consistently outperformed the rbf kernel which goes on to say the weight prediction data is linearly separable. Support vector machines for body weight prediction using default parameter kernel = rbf had the RMSE, MAE, R^2^ and correlation for the FS dataset were 1.569, 1.005, 0.832 and 0.944 respectively, for the PCA+ FS dataset, they were 1.461, 1.012, 0.861 and 0.959 respectively while for PCA they were 1.538, 1.025, 0.834 and 0.956 respectively. The hyperparameter optimization revealed the best hyperparameters of ‘C’: 1000, ‘gamma’: 1, ‘kernel’: ‘linear’ for the FS dataset, ‘C’: 1000, ‘gamma’: 0.0001, ‘kernel’: ‘rbf’ for the PCA and FS dataset and ‘C’: 100, ‘gamma’: 0.001, ‘kernel’: ‘rbf’ for the PCA dataset. The best-trained model had the following parameters: C: 1000, gamma: 1 kernel: linear.

### Regression trees and random forests for bodyweight prediction

Hyperparameter tuning improved the prediction results with random search performing better than grid search for breeding value prediction for most predictions except FS where grid search gave the best correlation results. For bootstrap = TRUE and max features = auto for the search algorithms. The highest correlation (0.990) was obtained for the FS dataset with grid search. Without hyperparameters, the FS dataset performed best for regression trees. The FS dataset had the highest correlation when compared with other datasets with all algorithms. Hyperparameter tuning improved the prediction ability of the random forests (Table 2).

### Gradient boost

The feature selection (FS) dataset had the highest correlation coefficient for the gradient boost algorithm with or without hyperparameters. The training results for the algorithm are given in Table 3.

### Polynomial regression

The highest correlation was found for the FS dataset with the average correlation reaching up to 0. 901. The 1st-degree polynomial gave the best-fit model. The training results for the algorithm are given in Table 3. The MAE values for the PCA, FS and FS+PCA datasets were 1.096, 0.709 and 1.078 respectively.

**Table 2:** Results obtained from regression trees, random forests and gradient boost.

	PCA+FS					PCA					FS				
	Regression trees	Random forrests		Gradient boost		Regression trees	Random forrests		Gradient boost		Regression trees	Random forrests		Gradient boost	
Hyperparameters	Default	Grid search	Random search	Default	Grid search	Default	Grid search	Random search	Default	Grid search	Default	Grid search	Random search	Default	Grid search
RMSE	1.805	1.729	1.629	1.603	1.349	2.049	1.63	1.595	1.61	1.322	0.945	0.901	0.946	0.968	0.95
MAE	1.138	1.127	1.097	1.098	1	1.244	1.123	1.098	1.103	0.967	0.83	0.813	0.835	0.831	0.836
R^2^	0.825	0.8	0.834	0.843	0.899	0.751	0.839	0.853	0.855	0.901	0.956	0.96	0.956	0.953	0.955
Correlation coefficient	0.893	0.914	** 0.926**	0.925	0.957	0.858	0.922	0.925	0.921	**0.96**	0.984	0.99	0.989	0.986	**0.991**
MSE	0.767	0.83	0.821	0.86	−0.458	0.74	0.815	0.828	0.849	−0.435	0.938	0.938	0.94	0.939	−0.119
Max Depth		15	10		1		10	10		1		15	20		2
N estimators		20	23		500		20	23		1000		13	6		2000
Subsample					0.5					0.5					0.75

The highest values obtained are in bold.

### XGBoost

The FS dataset had the highest correlation coefficient for the testing dataset with the XGBoost algorithm. All values are indicated in Table 3. The time elapsed for running the algorithm was the greatest for the PCA+FS dataset. The wall times for the PCA, FS and FS +PCA datasets were 93 ms, 91 ms, and 511 ms respectively. Colsample bytree, learning rate, Max depth, Min child weight, N estimators and Subsample for the PCA dataset were 0.7, 0.05, 3, 5, 1000 and 0.5, for the FS dataset were 0.7, 0.1, 3, 3, 1000 and for the FS+PCA dataset, they were 0.7, 0.01, 5, 5, 1000, 0.5 and 0.7 respectively.

### K nearest neighbours

The highest correlation between true and predicted values was found for the FS + PCA dataset (Table 3). The PCA dataset had the highest n-neighbours using hyperparameter tuning. The N neighbours for the PCA, FS and FS +PCA datasets were 7,4,3 respectively.

### MARS for body weight value prediction

The predicted and true value correlation coefficient was 0.993 while applying multivariate adaptive regression splines. The highest correlation coefficient was found for the FS dataset. All values are indicated in Table 3.

**Table 3:** Results obtained from XGBoost, KNN, Polynomial regression and MARS.

	XgBoost			K nearest neighbors			MARS			Polynomial regression					
	PCA	FS	FS+PCA	PCA	FS	FS+PCA	PCA	FS	PCA +FS	PCA		PCA+FS		FS	
										Mean	Best	Mean	Best	Mean	Best
RMSE	1.503	0.956	1.488	1.768	1.548	1.406	1.12	0.904	1.173	3.902	1.081	3.772	1.178	1.639	0.915
MAE	1.047	0.842	1.038	1.189	1.09	1.05	0.891	0.813	0.914	1.363	0.871	1.316	0.93	1.055	0.812
R^2^	0.846	0.953	0.851	0.761	0.837	0.882	0.935	0.959	0.926	0.73	0.94	0.793	0.924	0.834	0.957
Correlation coefficient	0.954	**0.99**	0.956	0.917	0.941	**0.949**	0.977	**0.993**	0.974	0.79	0.979	0.844	0.974	0.901	**0.993**

The highest values obtained are in bold.

### Algorithm ranking

**Figure 3:**
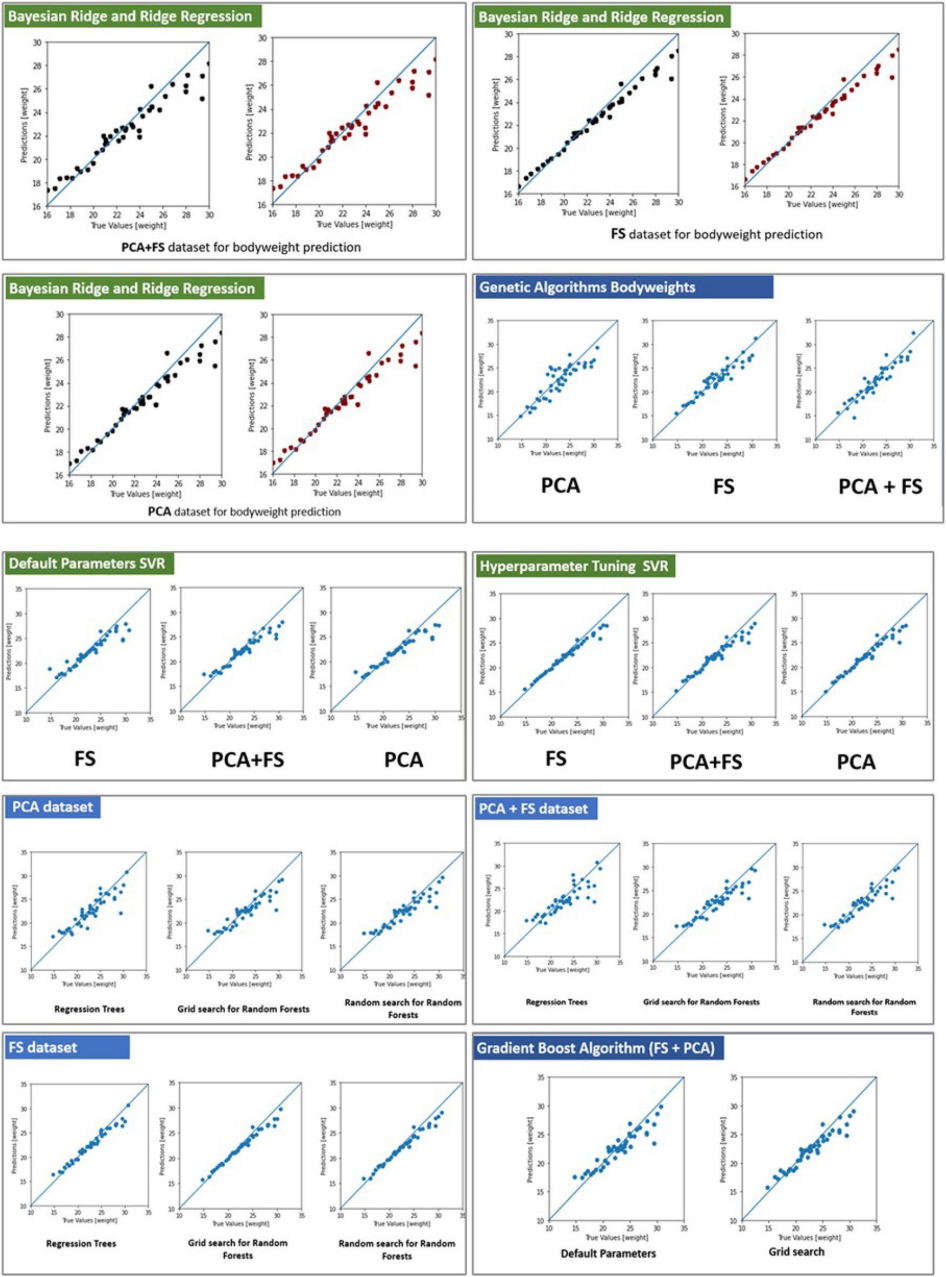
Pair plot for multicollinearity for the PCA+FS dataset.

**Figure 4:**
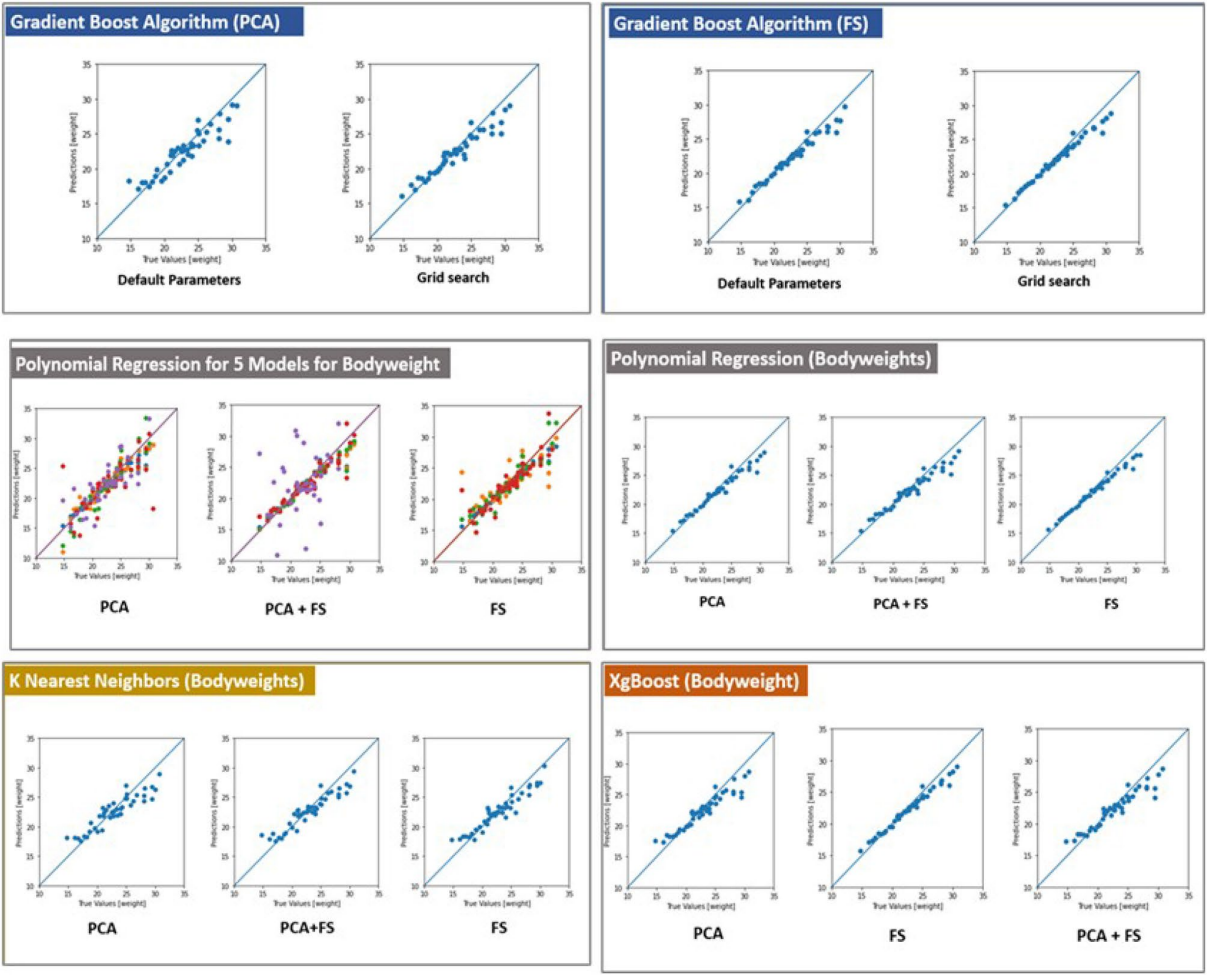
Correlation between true and predicted values of ML algorithms.

For the bodyweight prediction, the MARS algorithm gave the best predictions based on the correlation coefficient (Table 4) and for breeding value prediction, the tree-based algorithms gave the best results. Random forests had the highest correlation coefficient (Table 4). The FS dataset outperformed the PCA and PCA+FS datasets in most cases except for genetic algorithms and neural networks trained both by hyperparameter optimization as well as heuristic modelling and KNN (but only by a very narrow margin). For genetic algorithms, the dataset with the lowest number of features gave the best correlation coefficients. In the case of principal component regression, the PCA dataset performed best. Bayesian regression outperformed ridge regression by a small margin. The correlations between true and predicted values are given in Figs. 3 and 4.

**Table 4:** Ranking of algorithms for the prediction of body weights.

Rank	Name of the algorithm	Correlation coefficient
1	MARS algorithm	0.993
2	Bayesian ridge regression	0.992
3	Ridge regression	0.991
4	Support Vector Machines	0.991
5	Gradient boosting algorithm	0.991
6	Random forests	0.99
7	XgBoost algorithm	0.99
8	Artificial neural networks	0.984
9	Classification and regression trees	0.984
10	Polynomial regression	0.957
11	K nearest neighbours	0.949
12	Genetic Algorithms	0.734

## Discussion

Overall, all values that are to be taken at birth in the data were more meticulously recorded than the parameters that are to be recorded later in the life of the animal. Missing values are universal in real-world datasets and the use of winsorization to give the distribution more desirable statistical properties has also been published in literature for lowering the weight of influential observations and removing unwanted effects of outliers without the introduction of more bias. Anderson et al.^27^ converted a much higher range viz. the upper and lower 10% of data to the 90th percentile with a little introduction of error. A two-sided winsorization approach was used in this study which was also reported to be better than the one-sided approach by Chambers et al.^28^ and Hamadani et al.^29^.

The results of the present study indicate that the number of features was effectively reduced in the dataset using principal component analysis which substantially lowered the effective number of parameters characterizing the underlying model. The body weights taken at various ages from weaning had the greatest feature scores. This is expected as it is also evident from the growth curves of various animals in which body weight is the most important parameter^30^.

Feature selection has been shown by researchers to increase learning algorithms’ working both in terms of computation time and accuracy^31,32^. Our results of PCA reducing the multicollinearity to 1 correspond with the results of many authors^33,34^ as PCA has been reported in the literature as one of the most common methods to reduce multicollinearity in the dataset. The FS dataset had high multicollinearity as feature selection lessens the number of total features without dealing with the multicollinearity present within the dataset. It has been reported in the literature that multicollinearity does not affect the final model’s predictive power or reliability. The model predictions for ridge regression and Bayesian ridge similar to ours were reported by^19^ who also used various machine learning techniques for the prediction of weights and reported high R^2^ values approaching 0.988. A tenfold cross-validation for training the model was used which was also reported to be the most appropriate by^19^. However^35^, used 20-fold cross-validation for their study to predict breeding values.

A high coefficient of determination (0.92) was also stated by Kumar et al.^36^ and Adebiyi et al.^37^ for the estimation of weight from measurements and prediction of disease while^38^ reported R^2^ values of 0.70, 0.784 and 0.74 for the prediction of body weights in three Egyptian sheep breeds, Morkaraman sheep and in Malabari goats respectively. The R^2^ value, as well as the coefficient of correlation of the PCA dataset, was greater than the PCA+FS dataset from which it may be inferred that PCA is not just an effective technique for data reduction but also that further data reduction in the dataset caused some loss of variance in the dataset.

Compared to heuristic modelling, optimization algorithms took more time to execute. As the number of computations increases, they become increasingly difficult to solve and consume higher and higher computational power, sometimes even causing system crashes. This is so because optimization algorithms test a much higher number of options available to tune the best mode.

Our results indicate that all three datasets trained are comparable in terms of the correlation coefficient or training error. PCA+FS dataset converged earlier than the other two datasets upon hyperparameter tuning which may be because the number of features within this dataset is less than the other two and hence the convergence occurred earlier than the other two datasets. This is important for training efficiency especially when the datasets are large and the computational power available to the researcher is limited.

Out of the three datasets trained using both hyperparameter optimization and then by heuristic modelling, the PCA dataset showed the highest correlation coefficient of 0.977. From this one may infer that PCA efficiently took care of the selection of features that could sufficiently explain the variance of the data. FS alone performed better than the PCA+FS dataset which goes on to say that some of the explained variances may have been lost when both techniques were used together. The reduction in the number of features in this dataset alone was not enough to achieve the highest predictive ability of this dataset. Higher correlation for the prediction for the fat yield of 0.93 when predicted by ANN by Shahinfar et al.^39^. Peters et al. (2016) used the MLP-ANN model to achieve predictive correlations of 0.53 for birth weight, 0.65 for 205-day weight, and 0.63 for 365-day weight which is much lower than our prediction. Khorshidi-Jalali and Mohammadabadi^40^ compared ANNs and regression models for arriving at body weight in Cashmere goats and found the ability of the artificial neural network model to be better. However, unlike our results, this value was 0.86 for ANN.

Genetic algorithms performed poorly when compared to the other algorithms The lower-than-expected values may also be the reason that genetic algorithms are seldom used for direct regression. Genetic algorithms were also reported to be better suited for optimizing large and complex parametric spaces^41^.

For SVM the FS dataset had the highest correlation coefficient with the test labels and the hyperparameters for which the grid search was performed. The linear kernel consistently outperformed the rbf kernel suggesting that the weight prediction data is linearly separable. The rbf kernel has been reported to perform better in nonlinear function estimation by preventing noise to have a high generalization ability^42^. Ben-Hur et al.^43^ also observed that nonlinear kernels, Gaussian or polynomial, lead to only a slight improvement in performance when compared to a linear kernel. However, using a linear kernel, Long et al.^44^ reported a lower correlation coefficient of 0.497–0.517 for the prediction of quantitative traits. Alonso et al.^45^ also used 3 different SVR techniques for the prediction of body weights and reported higher prediction errors (MAE) of 9.31 ± 8.00, 10.98 ± 11.74, 9.61 ± 7.90 for the 3 techniques. Huma and Iqbal^19^also used support vector regression for the prediction of body weights in sheep and reported correlation coefficients, R^2^, MAE, and RMSE of 0.947, 0.897, 3.934, and 5.938 respectively which are close to the values in the present research.

Hyperparameter tuning improved the prediction results with random search performing better than grid search for breeding value prediction for most predictions except FS where grid search gave the best correlation results. Random search is very similar to grid search, yet it has been consistently reported to produce better results comparatively^46^ by effectively searching a larger, less promising configuration space.

Due to a difference in the relevance of hyperparameters for different models at hand, grid search sometimes becomes a poor choice for constructing algorithms for different data sets. Hyperparameters improved the prediction ability of the random forests which has also been published by^47,48^. Huma and Iqbal^19^ also used regression trees for the same prediction and reported R^2^ and MAE of 0.896, 4.583. They also used random forests for the prediction of body weights in sheep and reported correlation coefficients, R^2^, MAE, and RMSE of 0.947, 0.897, 3.934, and 5.938 respectively. When compared with other models. Many authors^19,49^ have stated the random forests method and their variants produce the lowest errors. Lower values for random forests (RF) were reported by Jahan et al.^50^ who reported an R^2^ of 0.911 for the bodyweight prediction of Balochi sheep. Çelik and Yilmaz^51^ also used the CART algorithm and reported lower values than the present study of R^2^ = 0.6889, Adj. R^2^ = 0.6810, r = 0.830 and RMSE = 1.1802, respectively. RF has also been suggested to be an important choice for modelling complex relationships between variables as compared to many other ML models for researchers based on its features. Similar to the results reported in the present study, random forests were also generally found to outperform other decision trees, but their accuracy was reported lower than gradient-boosted trees. Boosting algorithms are reported to perform well under a wide variety of conditions^52,53^. It is however important to mention that the convergence of algorithms also depends to a large extent on the data characteristics^54,55^.

Morphometric parameters along with body weights were used for the prediction of body weight with high correlation in this study. The highest variation of body weight was reported to be accounted for by the combination of chest girth, body length, and height for prediction of body weights by^56^.

XgBoost outperformed the gradient boost algorithm for the prediction of bodyweights. For the XGBoost algorithm, both the accuracy and the training speed were found to be better. This has also been published by Bentéjac et al.^57^ who compared XGBoost to several gradient-boosting algorithms. The XGBoost Algorithm was also shown to achieve a lower error value in comparison to random forests by Niang et al.^58^. XGBoost uses advanced regularization (L1 and L2), which may have been the reason for the improved model generalization capabilities^36^.

The greatest correlation was found for the FS + PCA dataset which means that through this technique a better prediction can be made using the least number of features. Support vector regression gave a slightly better convergence than k-nearest neighbours which was also stated by Ramyaa et al.^59^ in their study on phenotyping subjects based on body weight. KNN results have also been reported to be somewhat biased towards the mean with the extreme values of the independent variables but this did not affect the results of the present study.

The FS dataset gave the highest correlation coefficient using the multivariate adaptive regression splines algorithm. Again, the presence of a greater number of features than the other two datasets could have contributed to this. The R^2^ values closer to the ones obtained in this study of 0.972 obtained from the MARS algorithm for prediction of the fattening final weight of bulls were reported^60^. Çelik and Yilmaz^51^ used MARS for bodyweight prediction as well and reported slightly higher values of R^2^ equal to 0.919, RMSE equal to 0.604, and r equal to 0.959. MARS algorithm was reported to be a flexible model which revealed the interaction effects and minimized the residual variance^61^.

For the bodyweight prediction, the MARS algorithm gave the best predictions based on the correlation coefficient and for breeding value prediction, tree-based algorithms gave the best results. The FS dataset outperformed the PCA and PCA+FS datasets in most cases except for genetic algorithms and neural networks trained both by hyperparameter optimization as well as heuristic modelling and KNN (but only by a very narrow margin). This may be attributed to a greater number of features present within the FS dataset contributing to each causing the addition of some additional explained variance within the dataset towards the predicted variable. Bayesian regression outperformed ridge regression by a small margin going on to say that multicollinearity within the FS dataset did not cause any convergence issues which is also supported within the literature.

## Conclusion and recommendations

Artificial Intelligence is a promising area which has the potential to make accurate predictions about various aspects of farm management and can thus be a viable alternative to conventional strategies. 12 deployable and reusable models were developed in this study for the prediction of body weights at 12 months of age. All the models had high prediction ability with tree-based algorithms generally outperforming other techniques in regression-based tasks. These, if customized and deployed on farms, would help in taking informed decisions. Farm modernization would thus be beneficial for animal production, and the farm economy thus contributing to the larger goal of achieving food security.

## Methods

### Data preparation

To predict the body weight, data for 11 years (2011–2021) for the Corriedale breed was used and was collected from an organized sheep farm, in Kashmir. The total number of data points available for the study was 37201. Initial raw data included animal numbers (brand number, ear tag), dob, sex, birth coat, litter size, weaning date, parent record (dam number, sire number, dam weight, dam milking ability, parturation history), coat colour, time of birth, body weights (weekly body weights up to 4th week, fortnightly weights up to 6th fortnight, monthly body weight up to 12th month), monthly morphometric measurements up to weaning, weather data (daily temperature and humidity), disposal records, treatment records. Features were determined heuristically as well as using techniques discussed later. The raw data was cleaned, and duplicate rows with too many missing values were removed. Data imputation was done iteratively using Bayesian ridge regression^62^. Winsorization was used for handling outliers and the data were appropriately encoded and standardization was also done. This was achieved by dividing subtracting mean from each feature and dividing by the standard deviation. The data was split into training and testing, and the optimal train test split was heuristically determined with testing data equal to 10%, training data equal to 90 per cent of the dataset. The total training dataset was again for validation and the validation data proportioned to 10 per cent of the training data.

To decrease the number of input variables in the dataset and to select the ones contributing most to the variance, dimensionality reduction was performed using principal component analysis (PCA) and feature selection. PCA is a statistical technique which converts correlated features into a set of uncorrelated features linearly. This is done by orthogonal transformation. Feature selection was done in Python based on the F-test estimate of the degree of linear dependency between two numerical variables: the input and the output. Feature selection was performed both for the original datasets and after extracting features from PCA. The input variables were constant across all the ML methods used in this study so as to eliminate the bias that an uneven number of features/ input variables could cause during the training process. Thus, three datasets were created:

- The principal component analysis dataset (PCA) in which primarily the PCA technique was used for dimensionality reduction
- The feature selection dataset (FS) where the F-test estimate of the degree of linear dependency between two numerical variables was used for dimensionality reduction
- The PCA+FS dataset wherein both techniques were used to achieve a much-reduced number of features.
- Pure morphometric measurements were also used for predicting body weight using ANNs. This constituted the DM dataset which was used for the prediction of weaning weight. This was done because morphometric measurements were very scarce in the dataset after weaning.

Body weights at 12 months of age were used as labels. Weaning weight was also used as the label for one of the algorithms.

### Machine learning techniques

A total of 11 AI algorithms were employed in this study. Prediction of the weight parameter was done using body measurements as well as earlier body weights as input attributes to artificial neural networks. Hyperparameters were optimized using search-grid and random-search algorithms and later by heuristic tuning as well.

A comparison of the following machine learning algorithms was done in this study:

#### Bayesian ridge regression (BRR)^63^

This technique works on the principle that the output ‘y’ is drawn from a probability distribution and not a single value. Due to the inclusion of a probabilistic approach, the model is expected to train better. The prior for the coefficient “w” is thus derived using spherical Gaussian and the L2 regularization tested which is an effective approach for multicollinearity[10]. The cost function is a lambda term for a penalty to shrink the parameters thereby reducing the model complexity to get unbiased estimates. Default parameters of $1 {e^{-6}}$ for alpha 1 and alpha 2 were used. These are hyperparameters for the shape and rate parameters of the distribution.

#### Artificial neural networks^64^

This popular machine-learning technique is inspired by the neurons found in animal neural systems. A neural network is therefore only a group of units/nodes which are connected together to form artificial neurons[18]. This connection is similar to a neuron. Numbers just like signals in an actual brain are transmitted as signals among the artificial neurons and the output of each is calculated after a non-non-linearity is added to the sum of all inputs to that particular neuron. In a larger picture, the network of neurons is formed when many such neurons are aggregated into layers. The more the number of neurons, the denser is the neural network is formed. The addition of many inner layers is what makes the network deep. The hyperparameter ranges for PCA+FS, PCA and FS datasets respectively for Artificial Neural Networks were iterations = 1000, 200, 1000. Learning rate = 0.001, 0.5 for PCA +FS dataset, 0.001, 0.5 for PCA dataset, 0.001, 0.5 for FS dataset. Dropout rate = 0.01, 0.9 for PCA +FS dataset, 0.01, 0.9 for PCA dataset, 0.01, 0.9 for FS dataset. The hidden layers for the PCA+FS dataset = 1–5, PCA dataset = 1–7, and FS dataset =1–10. The neurons per layer for the PCA+FS dataset = 1300, PCA dataset = 1400, and FS dataset = 1400. The batch sizes per layer for the PCA+FS dataset = 8, 10, 16, 20, PCA dataset = 8, 10, 16, 20, 30, and FS dataset =8, 10, 16, 20, 30. The activation and optimizers options for datasets were ‘tanh’, ‘sigmoid’, ‘ReLU’ and ‘adam’, ‘rms’, and ‘sgd’.

#### Support vector machines^65^

This supervised machine learning algorithm (SVM) is useful for solving both regression (SVR) and classification (SVM) problems. SVM works by creating a maximum-margin hyperplane in the transformed input space. This way, the solution is optimized and a quadratic optimization problem is used to derive the hyperplane solution parameters. The grid search parameters for support vector machines with the ranges of Param grid $\copyright$ equal to 0.1, 1, 100, 10, 1000, gamma equal to 1, 0.1, 0.01, 0.001, 0.0001 and kernels equal to ‘rbf’, ‘sigmoid’, ‘linear’. A randomized search was conducted on the prespecified hyperparameters to estimate the best ones. The hyperparameter ranges for grid search and random search respectively were Bootstrap True and True, False, Max depth 5, 10, 20, 15, 30, None and 4 evenly spaced values between 5 and 20, max features equal to = ‘auto’, ‘log2’ and ‘auto’, ‘log2’, ‘sqrt’, n estimators equal to 5–13, 15, 20 and 20 evenly spaced values between 5 and 25.

#### Classification and regression trees algorithm (CART)^66^

CART algorithm works by building a decision tree. This decision tree works on Gini’s impurity index and uses it to arrive at a final decision. Analogous to an actual tree, each branching or fork represents a decision and the predictor variable is segregated towards either of the many branching points. And at the end, the end node arrives at the final target variable.

#### Random forests^67^

Random forests are similar to other tree-based algorithms. The theory, however, utilizes ensemble learning methods wherein many decision trees are constructed to arrive at a solution which is the most optimum. Thus the average of the prediction obtained from all such trees is taken as the final output.

#### Gradient boosting^54^

Again a tree-based ensemble algorithm utilizing many weak prediction decision trees. Thus the final model is built stage-wise. This allows the optimization of an arbitrary differentiable loss function which makes this algorithm better than many tree-based ones. The gradient boost algorithm hyperparameter options were learning rate = 0.001, 0.01, 0.1, N estimators = 500, 1000, 2000, subsample = 0.5, 0.75, 1, max depth = 1, 2, 4, and Random state = 1.

#### XGBoost^68^

Also a decision-tree-based algorithm making use of gradient boosting frameworks for arriving at the most optimum solutions. XGBoost uses extra randomization parameter, penalization of trees, proportional shrinkage of leaf nodes as well as newton boosting. Hyperparameter tuning for XGBoost grid search was taken as learning rates = 0.001, 0.01, 0.05, 0.1, Max depths = 3, 5, 7, 10, 20, Min child weight = 1, 3, 5, Subsample= 0.5, 0.7, Colsample by tree = 0.5, 0.7, N estimators= 50, 100, 200, 500, 1000 and Objective = ‘reg: squared error.

#### Polynomial regression^69^

Polnominal regression takes monomial regression a step ahead because here, the relationship between independent and dependent variables is represented as the nth-degree polynomial. This technique is useful for non-linear relationships between the dependent and independent variables. 10 degrees of polynomials were checked for the polynomial regression with a mean of 6 for each algorithm. Polynomial regression was implemented using the sklearn package in Python. The best parameters for the algorithm were derived using hyperparameter tuning as well.

#### K nearest neighbours^70^

A simple and effective machine learning algorithm which is a non-parametric learning classifier. It uses proximity for predicting data points. The assumption is that similar points would be close to each other on a plot and thus a predicted value is taken as the average of the n number ( k nearest neighbours) of points similar to it. that points that are similar would be found close to each other. Grid search was employed for KNN with the range of 2–11.

#### Multivariate adaptive regression splines (MARS)^71^

MARS combines multiple simple linear functions to aggregate them by forming the best-fitting curve for the data. It combines linear equations into an aggregate equation. This is useful for situations where linear or polynomial regression wouldn’t work. MARS algorithm was also used for all three datasets’ K-fold cross-validation. 10 splits and 3 repeats were used.

#### Genetic algorithms^72^

Techniques that solve constrained and unconstrained optimization problems as they are heuristic adaptive search algorithms belonging to the larger class of evolutionary algorithms. Being inspired by natural selection and genetics, genetic algorithms simulate the “survival of the fittest” among individuals of each generation for solving a problem. Each generation consists of a population of individuals all of whom represent points in search space.

### Evaluation metrics

For the model evaluation, four scoring criteria were used. And since the task at hand was a regression, these were mean squared error (MSE) given in Eq. 1, mean absolute error (MAE) given in Eq. 2, coefficient of determination (R^2^) presented in Eq.,3, and correlation coefficient $r$ represented in Eq. 4.

1 $$\begin{aligned} \hbox {MSE}= \sum\limits_{i=1}^{D}(x_i-y_i)^2 \end{aligned}$$

2 $$\begin{aligned} \hbox {MAE}= \sum\limits_{i=1}^{D}|x_i-y_i| \end{aligned}$$

3 $$\begin{aligned} R^2= & {} r^2 \end{aligned}$$

4 $$\begin{aligned} r= & {} \frac{\text {cov}(X,Y)}{\sigma _x \sigma _y} \end{aligned}$$

Here y_i_ equals the actual value for ith observation, x_i_ is the calculated value for ith observation and n represents the total number of observations.

## Data Availability

The data analysed during the current study are not publicly available because the authors do not have permission to share them publicly but are available from the corresponding author on reasonable request.
